# The Influence of Familial Predisposition to Cardiovascular Complications upon Childhood Obesity Treatment

**DOI:** 10.1371/journal.pone.0120177

**Published:** 2015-03-10

**Authors:** Louise A. Nielsen, Christine Bøjsøe, Julie T. Kloppenborg, Cæcilie Trier, Michael Gamborg, Jens-Christian Holm

**Affiliations:** 1 The Children’s Obesity Clinic, Department of Paediatrics, Copenhagen University Hospital Holbæk, DK 4300, Holbæk, Denmark; 2 Department of Paediatrics, Copenhagen University Hospital Herlev, DK 2730, Herlev, Denmark; 3 The Novo Nordisk Foundation Center for Basic Metabolic Research, Section of Metabolic Genetics, Faculty of Health and Medical Sciences, University of Copenhagen, DK 2200, Copenhagen, Denmark; 4 Institute of Preventive Medicine, Bispebjerg and Frederiksberg Hospitals, The Capital Region, DK 2000, Frederiksberg, Copenhagen, Denmark; 5 University of Copenhagen, Faculty of Health and Medical Sciences, DK 2200, Copenhagen, Denmark; University of Leipzig, GERMANY

## Abstract

**Introduction:**

The aim was to investigate whether a familial predisposition to obesity related cardiovascular complications was associated with the degree of obesity at baseline and/or changes in the degree of obesity during a multidisciplinary childhood obesity treatment program.

**Methods:**

The study included 1421 obese children (634 boys) with a median age of 11.5 years (range 3.1–17.9 years), enrolled in treatment for 0.04 to 5.90 years (median 1.3 years) at the Children's Obesity Clinic, Denmark. At baseline, weight and height were measured, body mass index (BMI) standard deviation score (SDS) calculated, and self-reported information on familial predisposition to obesity, hypertension, type 2 diabetes mellitus (T2DM), thromboembolic events, and dyslipidaemia were obtained. A familial predisposition included events in biological parents, siblings, grandparents, uncles, and aunts. The treatment outcomes were categorically analysed according to the prevalence of familial predispositions.

**Results:**

The median BMI SDS at enrolment was 3.2 in boys and 2.8 in girls. One-thousand-and-forty-one children had obesity in their family, 773 had hypertension, 551 had T2DM, 568 had thromboembolic events, and 583 had dyslipidaemia. Altogether, 733 had three or more predispositions. At baseline, familial T2DM was associated with a higher mean BMI SDS (*p* = 0.03), but no associations were found between the other predispositions and the children's degree of obesity. During treatment, girls with familial obesity lost more weight, compared to girls without familial obesity (*p* = 0.04). No other familial predispositions were associated with changes in BMI SDS during treatment.

**Conclusion:**

Obese children with a familial predisposition to T2DM showed a significantly higher degree of obesity at baseline and girls with familial obesity responded better to treatment. Besides these findings, no other associations were found between the occurrence of familial predispositions and the degree of obesity or changes herein during multidisciplinary childhood obesity treatment.

## Introduction

Through the past few decades, the prevalence of obesity in children and adolescents has increased though new studies suggest that the prevalence may have reached a plateau; nevertheless, obesity represents a serious challenge to the public health worldwide [1,2]. Childhood obesity is associated with severe comorbidities in regards to metabolic complications, such as dyslipidaemia, hypertension, insulin resistance, and prediabetes [3–6]. Furthermore, obese children are at risk of developing coronary heart disease and remaining obese as adults [3,7].

In order to treat childhood obesity effectively, an early intervention is of great importance, and a multidisciplinary approach involving the family combining counselling on diet, behaviour, and activity seems pivotal, but the response to treatment may be inadequate for some patients [8–13]. One explanation may be the metabolic state of the children and their families, since it has been shown that a familial predisposition to obesity is associated with a poorer response to treatment [9,10,12,14]. Furthermore, a familial predisposition to obesity has been associated with the development of obesity in childhood [15–18]. The influence of a familial predisposition to obesity has been described in regards to parents [9,11,12,14–16] especially the weight status of the mother [9,11,15], siblings [10], and grandparents [17,18] indicating that the high prevalence of overweight and obese children is dependent upon obesity among the family members.

The knowledge regarding the influence of familial type 2 diabetes mellitus (T2DM) and cardiovascular disease (CVD) upon the degree of obesity in later generations is less established. At present, the literature outlines that adolescents with T2DM show a tendency of being obese and having a familial predisposition to T2DM [19], but this particular familial predisposition has not been shown to influence the treatment outcome in obese children [12]. In addition, parental T2DM and hypertension have been associated with increased weight and skinfold thickness in a large cohort of children [20], whereas another study shows that children with a family history of CVD are more obese and that children suffering from metabolic complications also exhibit a higher prevalence of the very same complications in their families [21].

The present study investigated the association between body mass index (BMI) standard deviation score (SDS) and a familial predisposition to obesity, hypertension, T2DM, thromboembolic events, and dyslipidaemia in obese children and adolescents enrolled in a multidisciplinary childhood obesity treatment program. The study investigated whether these familial predispositions influenced the degree of obesity at baseline or the changes in BMI SDS during treatment.

## Materials and Methods

### Study population

This prospective study was based on 1732 children and adolescents enrolled in treatment at The Children’s Obesity Clinic, Department of Paediatrics, Copenhagen University Hospital Holbæk, Denmark. The children had a BMI above the 90^th^ percentile adjusted for age and gender and were enrolled in the clinic from January 2008 to January 2014. Of the 1732 children, 1421 children met the inclusion criteria being between 3–18 years of age and having a record of at least two anthropometric measurements; at baseline and at a later time point during treatment, besides registration of familial predispositions. There were no other exclusion criteria.

### Baseline

At baseline, anthropometric measurements and a substantial medical history were obtained. The medical history included self-reported familial predispositions to obesity, hypertension, T2DM, thromboembolic events, and dyslipidaemia.

#### Anthropometry

Anthropometric measurements were used to assess the patients’ degree of obesity. The height was measured to the nearest 0.1 cm using a stadiometer. The weight was measured to the nearest 0.1 kg using a Tanita Quality Control Seal, WB-100 MA. The patients wore light indoor clothes with empty pockets and no shoes during measurements. BMI was calculated as the weight in kilograms divided by the square of the height in meters (kg/m^2^). Using the LMS method, which compares the calculated BMI with the distribution of BMI in a Danish standard population adjusted for age and gender, the BMI SDS was calculated [22]. BMI SDS is an accepted and available measure of adiposity in children and it is normal distributed in this population, please refer to the “Supporting Information”.

#### Familial predisposition

The presence of a familial predisposition was assessed by asking the patients and parents whether any of the biological family members up to and including second-degree relatives (parents, siblings, grandparents, uncles, and aunts) have or have had obesity, hypertension, T2DM, thromboembolic events, or dyslipidaemia. The questions were asked using Danish layman’s language and were initially addressed for maternal predispositions, in 5 separate questions, followed by paternal predispositions, in order to avoid confusion of maternal and paternal familial predispositions. The designation of a familial predisposition required at least one case in the family.

### Intervention

All patients were enrolled in a multidisciplinary childhood obesity treatment program conducted by paediatricians, nurses, dieticians, social workers, and psychologists [13]. Every patient received a tailored treatment plan with 10–20 advices which the patient and their family had to follow in order to reduce the degree of obesity in the child or adolescent [13]. These advices seek to overcome unfavourable food choices, eating disturbances, excessive sugar and fat intake, malnutrition, sedentary behaviour, sleep deprivation, and more [13]. At each consultation, anthropometric measurements were obtained, and the treatment plan was adjusted in order to attain weight loss or sustain weight maintenance [13]. The patients visited The Children’s Obesity Clinic with a mean frequency of six weeks, and the healthcare professionals spend an average of 5.4 hours per child per year [13]. For a more concise description of the treatment protocol, please refer to [13].

### Statistical analysis

The patients were categorized according to whether they had any of the following familial predispositions: Obesity, hypertension, T2DM, thromboembolic events or, dyslipidaemia. In each group the predisposed patients were compared with the non-predisposed patients. The patients were furthermore categorized according to accumulated familial predispositions, in which patients with three or more familial predispositions, i.e. the occurrence of at least 3 of the 5 different familial predispositions, were compared to patients with 2 or less familial predispositions. During the analyses, we assumed that the individual exposure of familial predisposition had a constant influence on the rate of weight loss during treatment.

In order to determine whether a familial predisposition influenced the degree of obesity at baseline, a regression analysis with baseline BMI SDS as outcome and the predisposition as an independent variable, adjusted for gender, was made for each of the groups. Afterwards, the treatment outcomes were evaluated for boys and girls separately. The longitudinal development of BMI SDS during treatment was modelled using a generalized linear mixed model. The covariance structure includes a random intercept and a random slope allowing each patient to have its own overall level and development of BMI SDS, and an exponential residual structure, allowing the covariance between two measurements on the same patient to decrease as the time between measurements increases. The mean value of BMI SDS was modelled as a function of time since treatment initiation, using a cubic spline with three a priori-chosen knots (at 2, 7, and 16 months, respectively). By using this model, the association between BMI SDS and time were allowed to be non-linear. The model included a thorough modelling of the variance structure in order to prevent bias originating from drop-out. For further description of the model, please refer to [13]. For a graphic presentation of the data including probability plots, residual plots, and variograms, which were used as model diagnostics, please refer to the “Supporting Information”. The associations between familial predisposition and treatment outcome are presented as estimates of differences in BMI SDS changes per year (ΔBMI SDS) with 95% confidence intervals. We applied a significance level of 5% (two-sided).

### Ethics statement

Informed written consent was obtained from the patients aged 18 years or from their legal guardians when younger. The study was ethically approved by the Ethics Committee of Region Zealand, Denmark (protocol no. SJ-104) and by the Danish Data Protection Agency. The study was executed in accordance with the ethical standards of the Declaration of Helsinki 2013.

## Results

### Baseline

The 1421 included patients had a median age of 11.5 years (range 3.1–17.9 years), 634 were boys and they were followed for a median period of 1.33 years (0.04–5.90). All median baseline characteristics are listed in Table 1. At the onset of treatment, 1041 obese children and adolescents had a familial predisposition to obesity (73%), 773 to hypertension (54%), 551 to T2DM (39%), 568 to thromboembolic events (40%), 583 to dyslipidaemia (41%), and 733 had three or more predispositions (52%), (Table 2). A familial predisposition to T2DM was significantly associated with a 0.078, 95% CI [0.01; 0.15] higher mean BMI SDS at baseline (*p* = 0.025) compared to children with no familial predisposition to T2DM, (Table 2). No associations were found between the degree of obesity at baseline, and a familial predisposition to obesity (*p* = 0.16), hypertension (*p* = 0.80), thromboembolic events (*p* = 0.75), dyslipidaemia (*p* = 0.54), or three or more predispositions (*p* = 0.21), (Table 2).

**Table 1:** Baseline characteristics and median follow-up time.

	Total median (range)	Boys median (range)	Girls median (range)
**N**	1421	634	787
**Age (years)**	11.5 (3.1–17.9)	11.8 (3.3–17.9)	11.3 (3.1–17.9)
**Height (cm)**	152.7 (97.0–194.5)	154.0 (99.2–194.5)	152.0 (97.0–180.5)
**Weight (kg)**	62.1 (18.9–185.0)	63.2 (18.9–155.2)	60.9 (19.4–185.0)
**BMI SDS^1^**	2.96 (1.65–6.12)	3.22 (1.72–6.12)	2.80 (1.65–5.48)
**Follow-up (years)**	1.33 (0.04–5.90)	1.32 (0.04–5.80)	1.34 (0.04–5.90)

^1^BMI SDS, body mass index standard deviation score.

**Table 2:** The prevalence of familial predispositions in obese children and adolescents and the association between familial predispositions and the degree of obesity at baseline.

	N	%	Disposed vs. non-disposed [95% CI]^1^	*p*
**Obesity**	1041	73	0.05 [−0.02;0.13]	0.16
**Hypertension**	773	54	0.01 [−0.06;0.08]	0.80
**T2DM**	551	39	0.08 [0.01;0.15]	0.025*
**Thromboembolic events**	568	40	0.01 [−0.06;0.08]	0.75
**Dyslipidaemia**	583	41	0.02 [−0.05;0.09]	0.54
**Three or more dispositions**	733	52	0.04 [−0.02;0.11]	0.21

^1^Difference in mean BMI SDS level between disposed and non-disposed adjusted for gender

*Significant association, *p*<0.05

### Treatment outcome

The response to treatment was analysed for boys and girls separately. In boys, the mean BMI SDS at baseline was 3.24, 95% CI [3.18;3.29] and 2.89, 95% CI [2.82;2.95], (ΔBMI SDS-0.35) after one year of treatment (Table 3). The girls had a mean BMI SDS of 2.82, 95% CI [2.78;2.86] at baseline and 2.61, 95% CI [2.56;2.65], (ΔBMI SDS-0.21) after one year of treatment (Table 3).

**Table 3:** Treatment outcome after one year of treatment in obese children and adolescents.

	Mean Baseline BMI SDS [95% CI]	Mean BMI SDS after one year [95% CI]	ΔBMI SDS [95% CI]
**Boys**	3.24 [3.18;3.29]	2.89 [2.82;2.95]	−0.35 [−0.39;−0.31]
**Girls**	2.82 [2.78;2.86]	2.61[2.56;2.65]	−0.21 [−0.24;−0.19]

The influence of a familial predisposition to obesity, hypertension, T2DM, thromboembolic events, dyslipidaemia, and three or more predispositions on the treatment outcome was analysed separately for boys and girls. Familial predisposition to obesity in girls was significantly associated with changes in BMI SDS during treatment, since the mean BMI SDS decreased 0.038 more per year of treatment than in girls without obesity in their family (*p* = 0.041), (Table 4). No associations were found in girls in regards to changes in BMI SDS and a familial predisposition to hypertension, T2DM, thromboembolic events, dyslipidaemia, or three or more predispositions (Table 4). Furthermore, none of the familial predispositions were associated with changes in BMI SDS during treatment in boys (Table 4).

**Table 4:** The influence of familial predispositions and the response to treatment per year in obese children and adolescents.

	Boys		Girls	
	Disposed vs. non-disposed [95% CI]^1^	*p*	Disposed vs. non-disposed [95% CI]^1^	*p*
**Obesity**	−0.013 [−0.068;0.041]	0.629	−0.038 [−0.074; −0.002]	0.041*
**Hypertension**	−0.006 [−0.057;0.045]	0.818	−0.031 [−0.065;0.004]	0.086
**T2DM**	0.019 [−0.033;0.072]	0.464	−0.008 [−0.044;0.028]	0.67
**Thromboembolic events**	−0.004 [−0.056;0.047]	0.865	−0.027 [−0.063;0.010]	0.15
**Dyslipidaemia**	0.013 [−0.040;0.067]	0.626	−0.007 [−0.043;0.029]	0.70
**Three or more dispositions**	0.001 [−0.051;0.052]	0.975	−0.029 [−0.065;0.006]	0.10

^1^ΔBMI SDS. Difference in change in BMI SDS per year, negative values correspond to faster weight loss in the disposed group

*Significant association, *p*<0.05.

## Discussion

The present study hypothesised that the occurrence of a familial deranged metabolic state, as an inherited or environmental component, could potentially affect the treatment outcome of obese children and adolescents enrolled in a childhood obesity treatment program. The study showed that children with a familial predisposition to T2DM have a higher degree of obesity at treatment initiation.

A similar tendency has been shown in a small cohort of children with diabetic parents where children with parental T2DM had a higher BMI adjusted for age and gender compared to children without diabetic parents [23]. Since the knowledge in this field seem limited, further investigations needs to be elaborated in order to confirm this finding.

The present study also showed that after one year of treatment the boys had a 0.35 reduction in mean BMI SDS whereas the girls exhibited a 0.21 reduction in their mean BMI SDS. When grouped according to familial predispositions, girls with familial obesity responded better to treatment than girls with no familial obesity, though girls without familial obesity reduced their degree of obesity as well. This finding was contrary to expectations since other studies have shown that familial obesity is associated with a poorer response to treatment [9–12,14]. However, familial predispositions to obesity related cardiovascular complications were in general not associated with the changes in BMI SDS in the obese children and adolescents enrolled in the multidisciplinary treatment protocol practised in the present study. Furthermore, the rather small effect sizes seen in the associations between familial T2DM and obesity and BMI SDS at baseline and after one year of treatment respectively are potentially sensitive to adjustment for multiple testing, which underline the need for evaluation of the associations in future larger studies, which uses a more comprehensive definition of familial predisposition.

The primary strength of the present study is the large study population of 1421 obese children included in treatment and that no eligibility criteria, other than age and degree of obesity, were used. In the literature, a few intervention studies were identified, which all associated a familial predisposition to obesity with a poorer response to treatment. In these studies 77 [14], 111 [10], 112 [12], and, 643 [9] children were included in treatment. However, the majority of these studies excluded children with competitive diseases and use of medications from the treatment program, which makes the treatment groups slightly different from those of the present study. Further, considerations to ethnicity and geographical place of residence also create potential differences. Finally, the intervention approaches and the follow-up times during treatment varied from 12 [10,12,14] to 36 months [9] in-between these studies.

Furthermore, many of the cited studies outline the effect of a familial predisposition to obesity [9–12,14–18] and only a few studies outline the effect of a familial predisposition to T2DM [12]. The studies concerning CVD only analyse the degree of obesity and not how these conditions influence childhood obesity treatment [20,21]. Glowinska *et al* showed an association between a familial predisposition to CVD and BMI in a group of children suffering from obesity, hypertension, and type 1 diabetes mellitus [21]. The children were stratified according to their diseases and the BMI in the obese children was associated with a family history to premature CVD (CVD manifested before the age of 55) [21]. However, this subgroup consisted of only six children who furthermore were older than the controls, which may bias the results since they used BMI without adjustment for age and gender, which is important due to growth and development during childhood. Additionally, some of the studies are restricted only to the patient’s parents, but since a familial predisposition is effected as a combination of an inherited as well as an environmental component, the general family history seems to be important [9,11–15,20].

The primary limitation of the present study lies within the questionnaire used at enrolment. If the patients and their parents could not recall the exact existence of familial predispositions, the paediatrician may then neglect potential information and thus misclassify the familial predispositions. Therefore, the results may harbour an inaccurate estimate of the prevalence of familial predispositions due to recall bias, especially in families with a single parent or in consultations in which only one of the parents attended. Furthermore, the present study did not take the size of the families into account, which is important, since the occurrence of familial predispositions may be more prevalent in larger families and vice versa. The same limitation exists within the age of the family members since relatively older parents and related family members exhibit a relatively greater probability of developing obesity, T2DM, or CVD.

For future studies, it would be interesting to stratify the children according to their obesity related complications and more precisely investigate from whom and from how many family members the familial predispositions arose, since it may contribute to the identification of patients with less efficient treatment responses and thus allow the clinicians to intensify treatment in these particular subgroups. Finally, analyses of familial predispositions combined with metabolomics and genetics presumably will strengthen future studies.

## Conclusions

The present study found that a familial predisposition to T2DM was associated with a higher degree of obesity at baseline. Furthermore, girls with a familial predisposition to obesity responded better to childhood obesity treatment. In general, changes in BMI SDS during multidisciplinary childhood obesity chronic care treatment were not influenced by familial predispositions to obesity and/or related cardiovascular complications.

## Supporting Information

S1 Fig Treatment Outcome (Boys). The distribution of changes in BMI SDS during treatment for each individual boy.
(PDF)

S2 Fig Treatment Outcome (Girls). The distribution of changes in BMI SDS during treatment for each individual girl.
(PDF)

S3 Fig Marginal Distribution (Boys). A histogram of the distribution of marginal BMI SDS residuals in boys, showing to be normal distributed among the boys.
(PDF)

S4 Fig Marginal Distribution (Girls). A histogram of the distribution of marginal BMI SDS residuals in girls, showing to be normal distributed among the girls.
(PDF)

S5 Fig Marginal Residual Plot (Boys). A plot of the marginal residuals in boys compared with the predicted values.
(PDF)

S6 Fig Marginal Residual Plot (Girls). A plot of the marginal residuals in girls compared with the predicted values.
(PDF)

S7 Fig Marginal Probability Plot (Boys). A plot comparing the sample quantiles with the theoretical quantiles of the marginal residuals in the boys.
(PDF)

S8 Fig Marginal Probability Plot (Girls). A plot comparing the sample quantiles with the theoretical quantiles of the marginal residuals in the girls.
(PDF)

S9 Fig Conditional Distribution (Boys). A histogram of the distribution of conditional BMI SDS residuals in boys, showing to be normal distributed among the boys.
(PDF)

S10 Fig Conditional Distribution (Girls). A histogram of the distribution of conditional BMI SDS residuals in girls, showing to be normal distributed among the girls.
(PDF)

S11 Fig Conditional Residual Plot (Boys). A plot of the conditional residuals in boys compared with the predicted values.
(PDF)

S12 Fig Conditional Residual Plot (Girls). A plot of the conditional residuals in girls compared with the predicted values.
(PDF)

S13 Fig Conditional Probability Plot (Boys). A plot comparing the sample quantiles with the theoretical quantiles of the conditional residuals in the boys.
(PDF)

S14 Fig Conditional Probability Plot (Girls). A plot comparing the sample quantiles with the theoretical quantiles of the conditional residuals in the girls.
(PDF)

S15 Fig Variogram (Boys). A plot of the half-squared differences between residuals over differences in time for the boys, with a smoothed curve showing that the covariance declines as time between measurements increases.
(PDF)

S16 Fig Variogram (Girls). A plot of the half-squared differences between residuals over differences in time for the girls, with a smoothed curve showing that the covariance declines as time between measurements increases.
(PDF)
