# Supplementary figures and images for: The Influence of Familial Predisposition to Cardiovascular Complications upon Childhood Obesity Treatment
Source: PLoS One. 2015 Mar 10;10(3):e0120177. doi: 10.1371/journal.pone.0120177 (PMC4355065; doi:10.1371/journal.pone.0120177)

# Boys

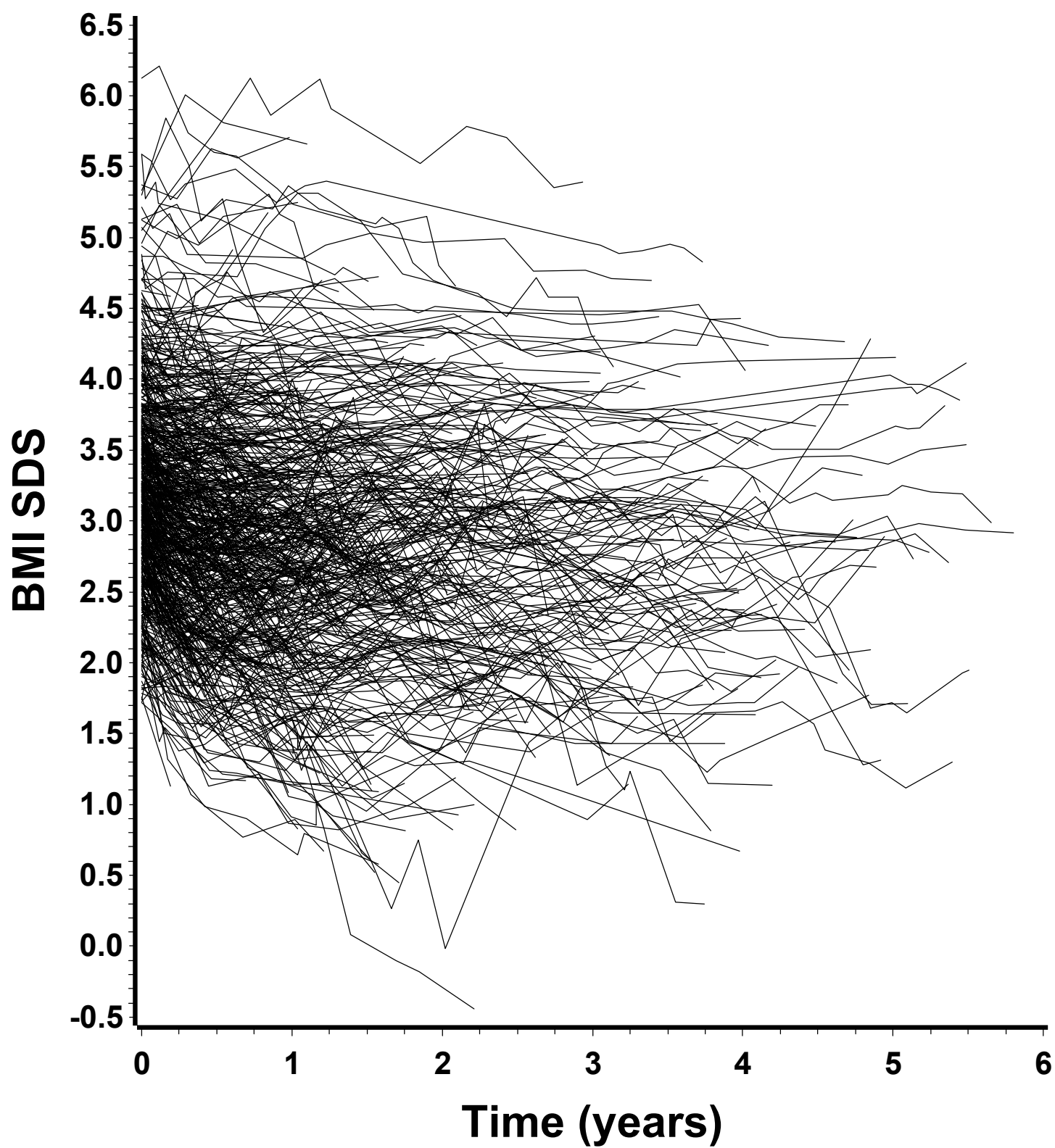

Supplement: S1 Fig — The distribution of changes in BMI SDS during treatment for each individual boy. (PDF) [file pone.0120177.s001.pdf]

# Girls

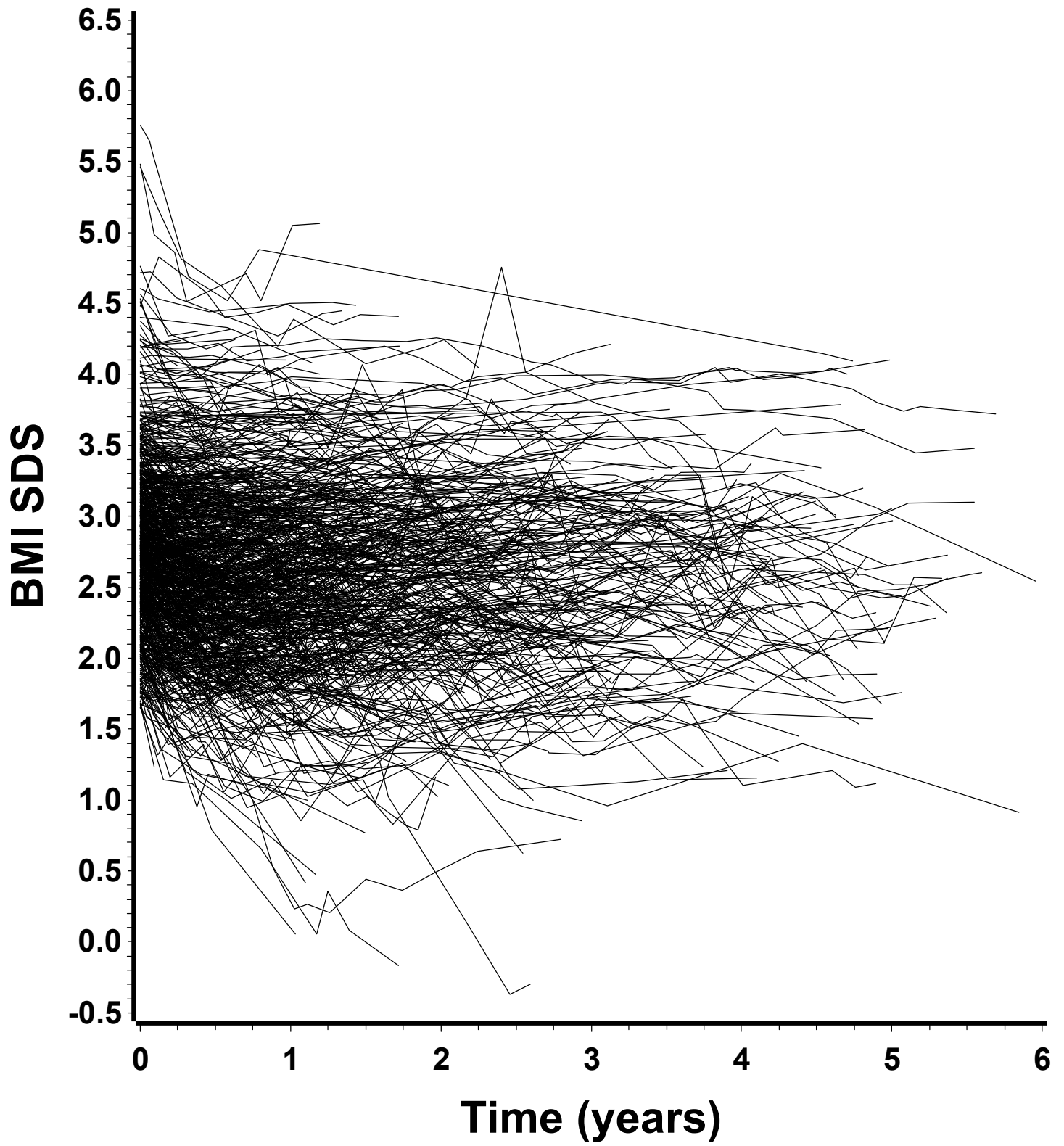

Supplement: S2 Fig — The distribution of changes in BMI SDS during treatment for each individual girl. (PDF) [file pone.0120177.s002.pdf]

# Boys

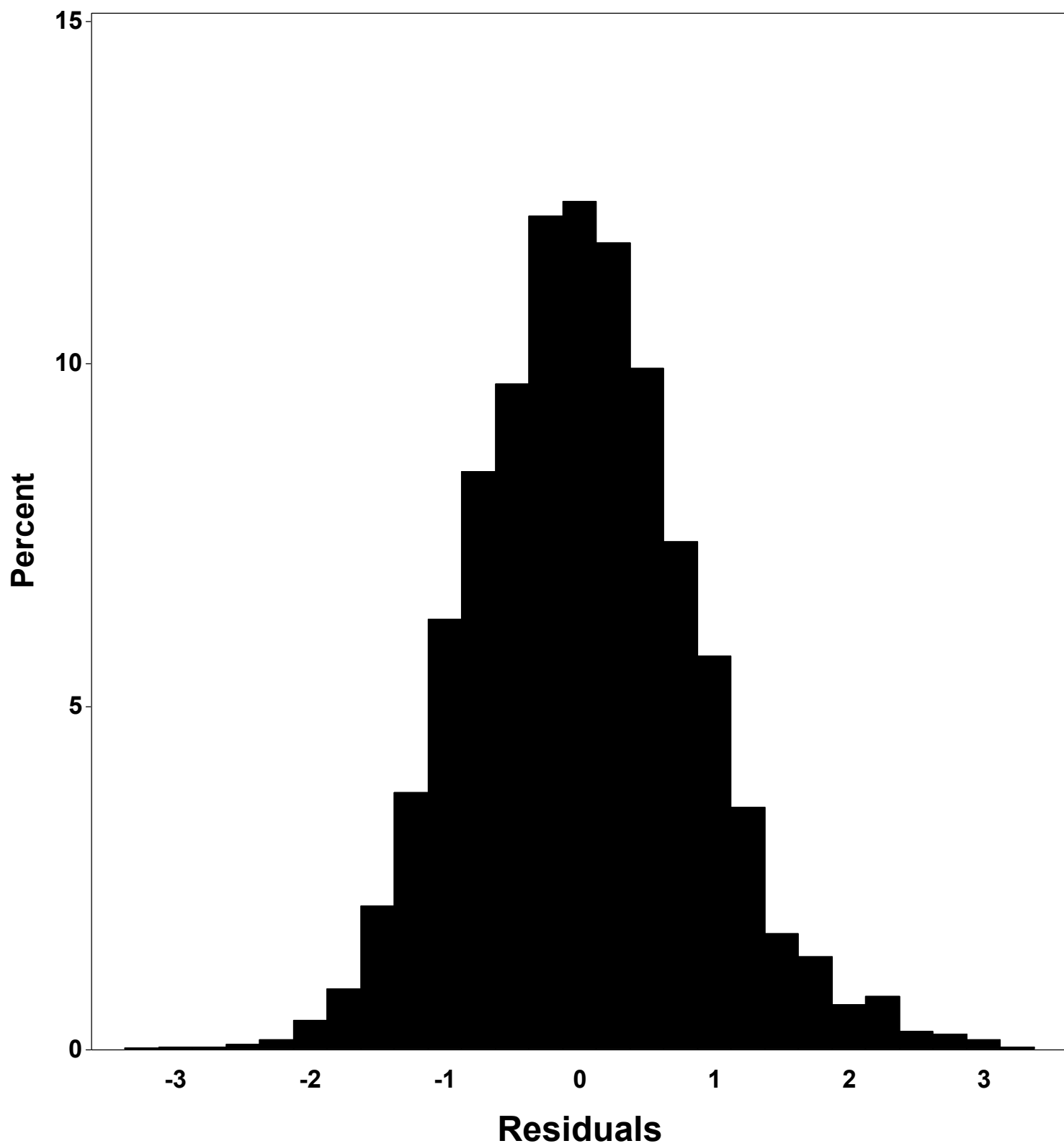

Supplement: S3 Fig — A histogram of the distribution of marginal BMI SDS residuals in boys, showing to be normal distributed among the boys. (PDF) [file pone.0120177.s003.pdf]

# Girls

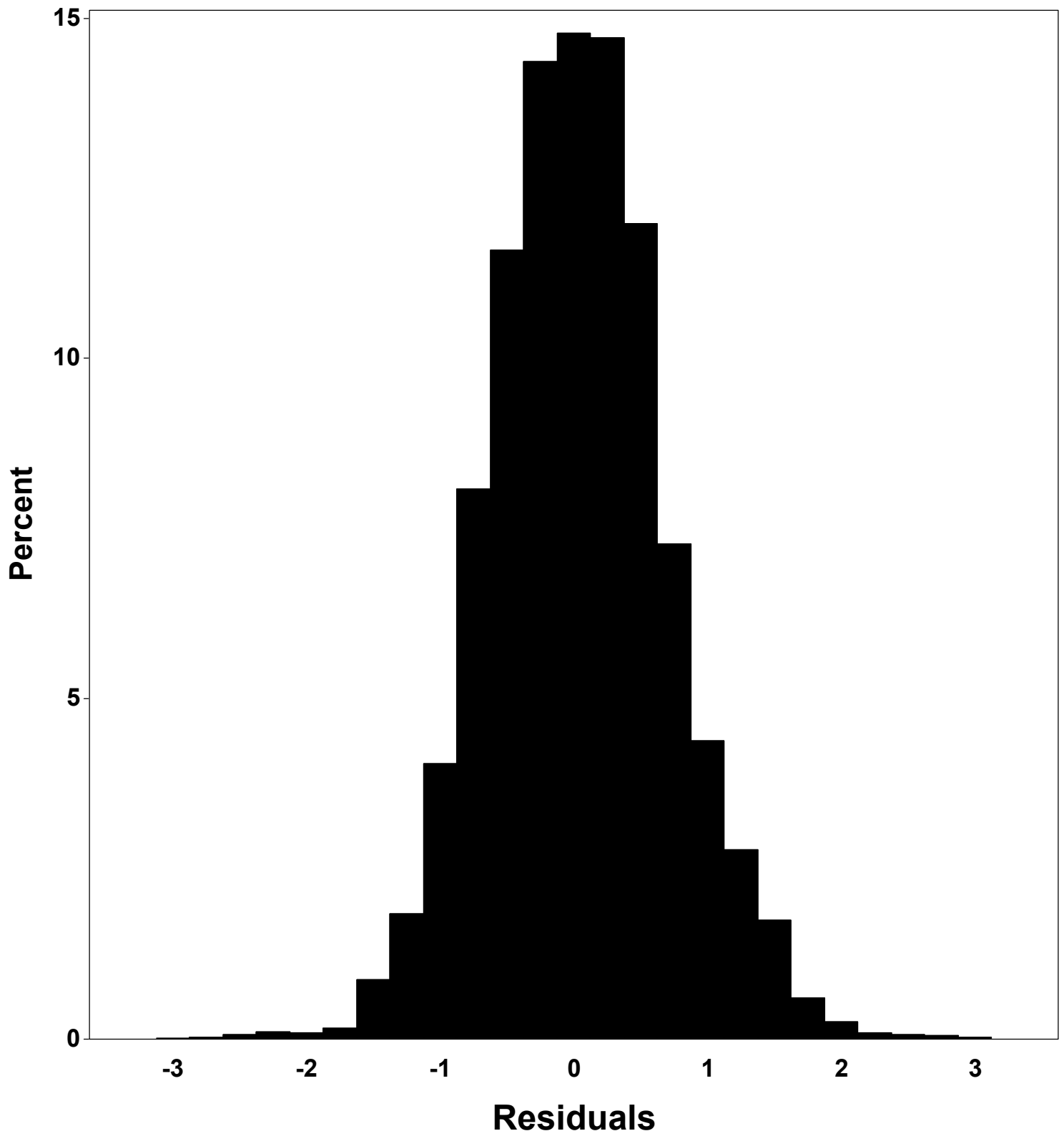

Supplement: S4 Fig — A histogram of the distribution of marginal BMI SDS residuals in girls, showing to be normal distributed among the girls. (PDF) [file pone.0120177.s004.pdf]

# Boys

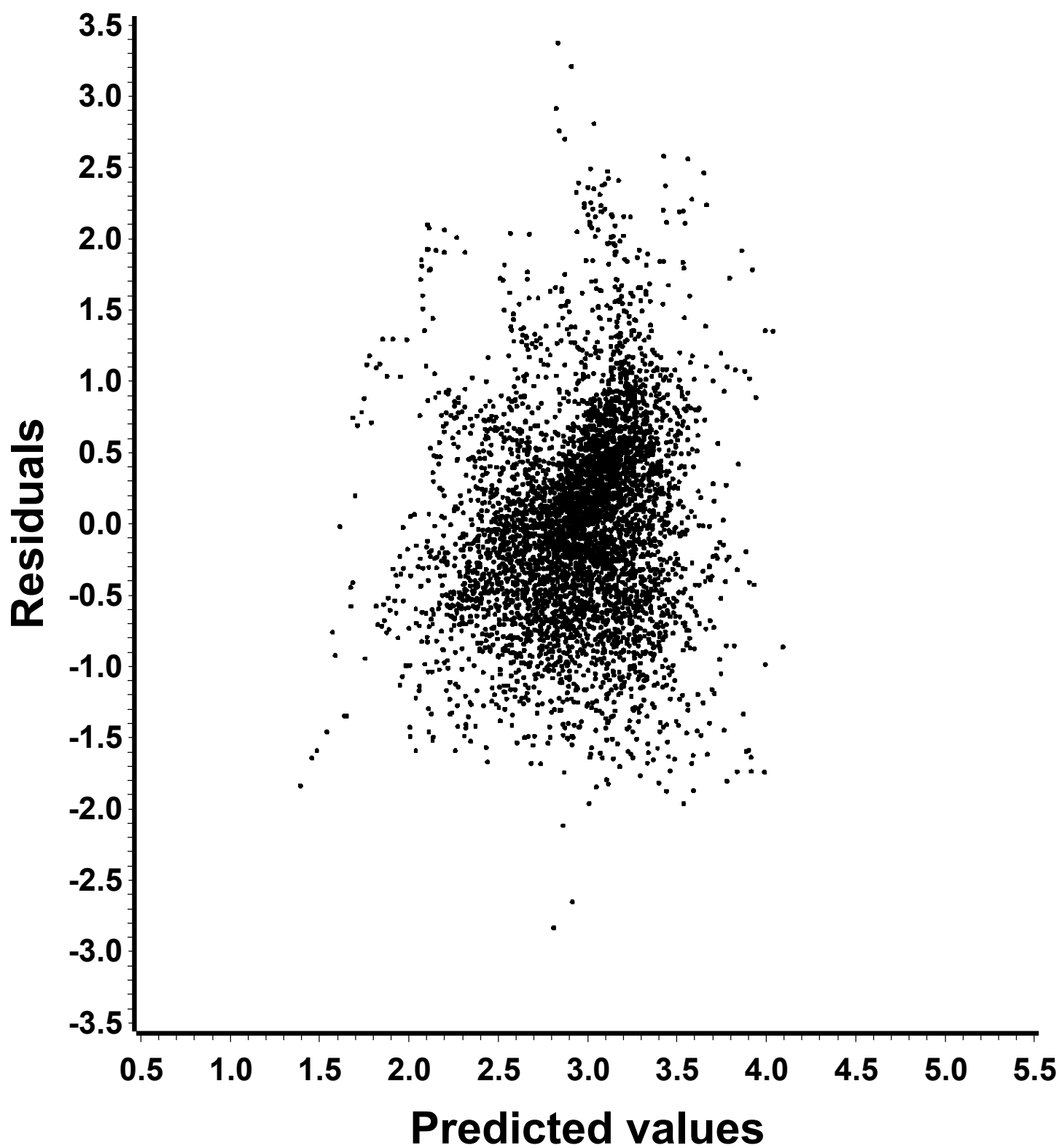

Supplement: S5 Fig — A plot of the marginal residuals in boys compared with the predicted values. (PDF) [file pone.0120177.s005.pdf]

# Girls

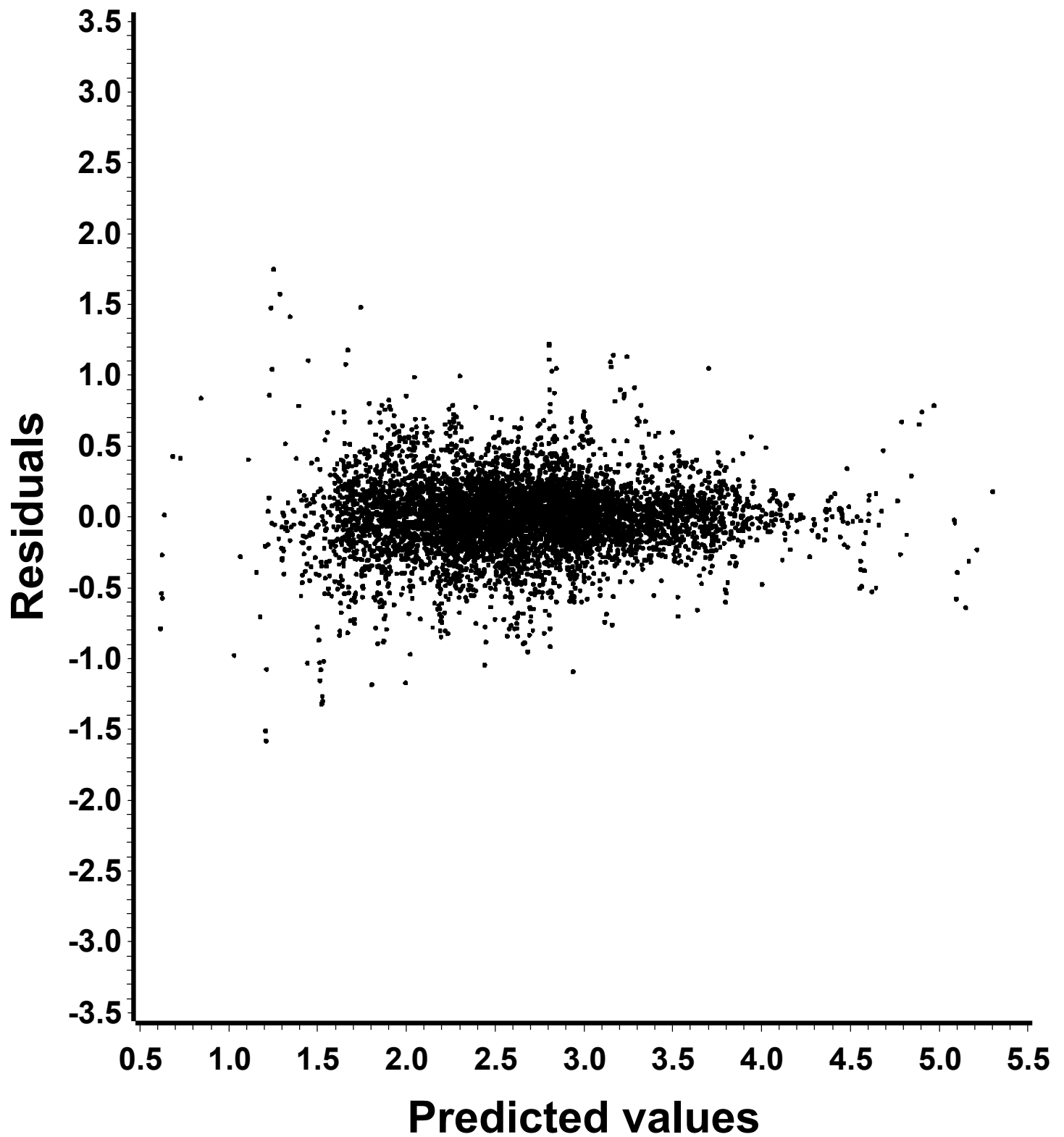

Supplement: S6 Fig — A plot of the marginal residuals in girls compared with the predicted values. (PDF) [file pone.0120177.s006.pdf]

# Boys

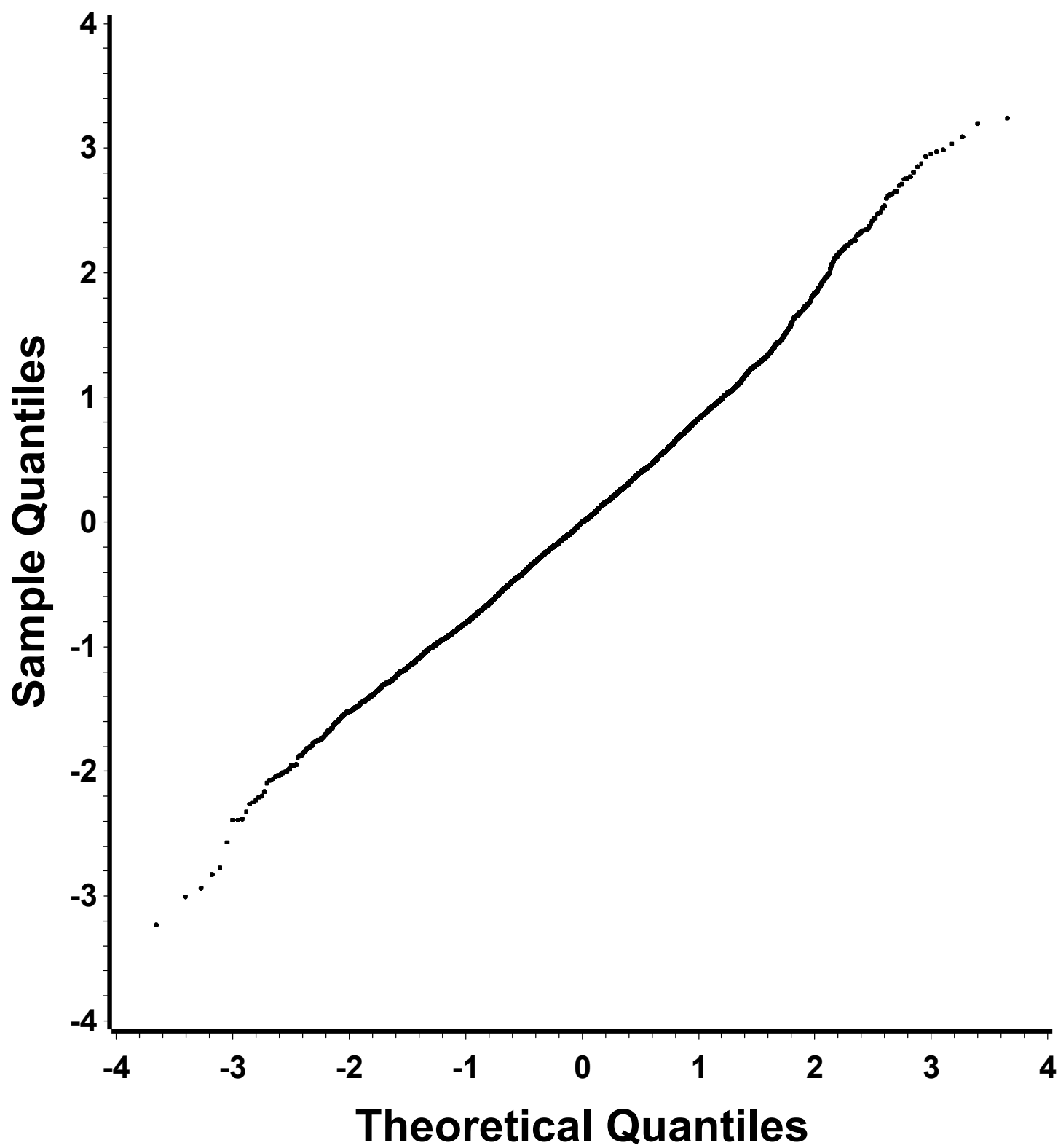

Supplement: S7 Fig — A plot comparing the sample quantiles with the theoretical quantiles of the marginal residuals in the boys. (PDF) [file pone.0120177.s007.pdf]

# Girls

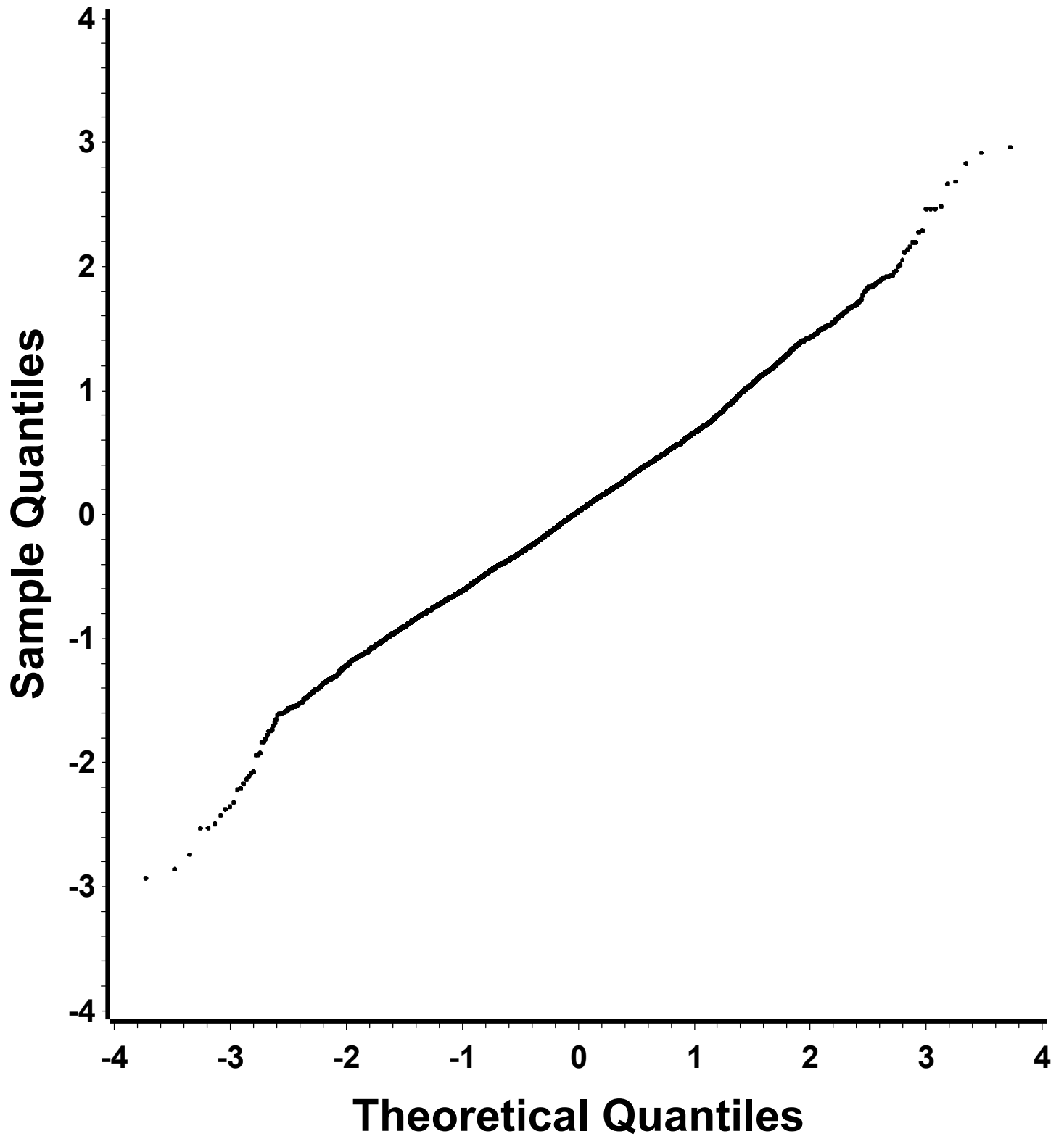

Supplement: S8 Fig — A plot comparing the sample quantiles with the theoretical quantiles of the marginal residuals in the girls. (PDF) [file pone.0120177.s008.pdf]

# Boys

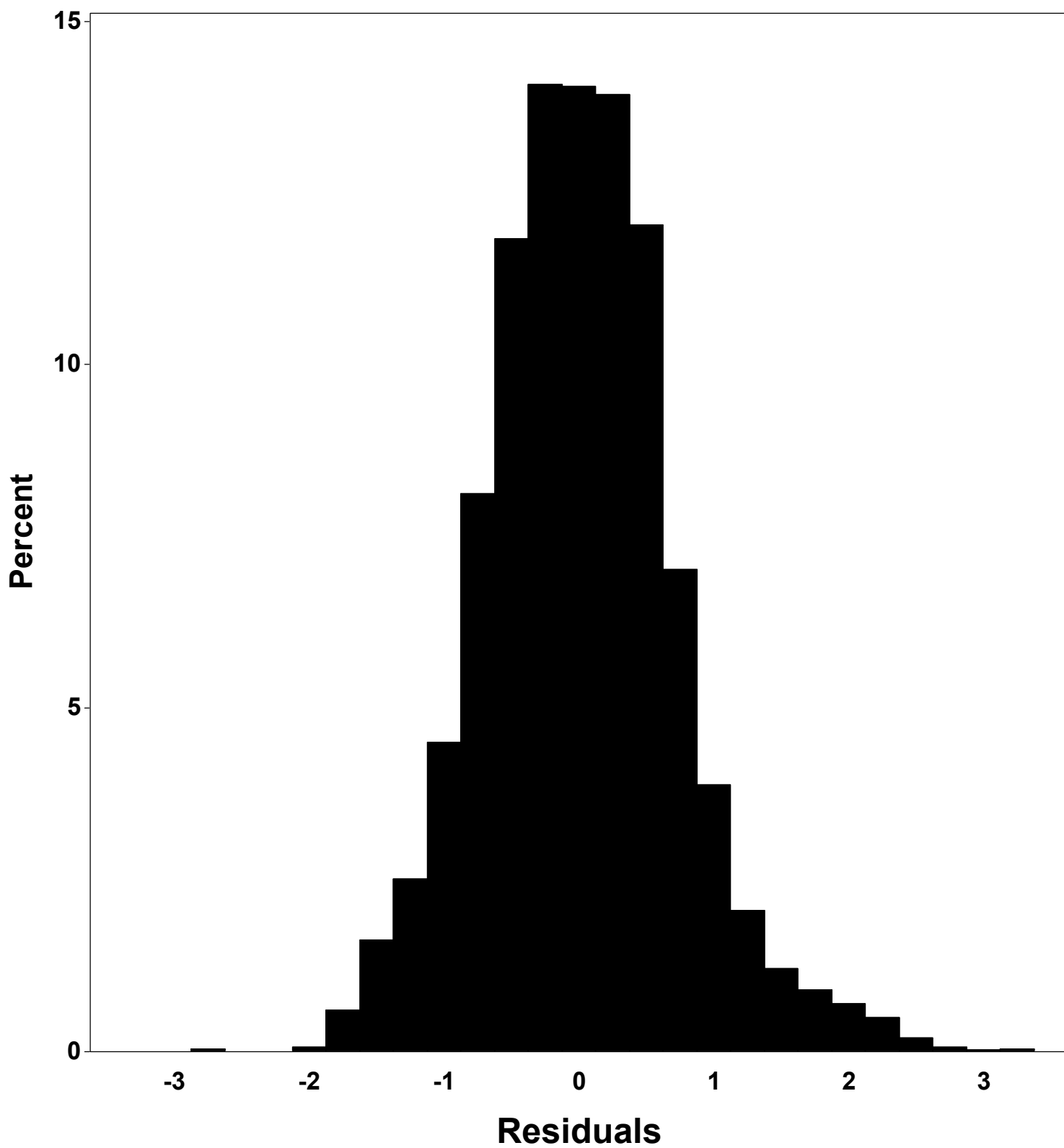

Supplement: S9 Fig — A histogram of the distribution of conditional BMI SDS residuals in boys, showing to be normal distributed among the boys. (PDF) [file pone.0120177.s009.pdf]

# Girls

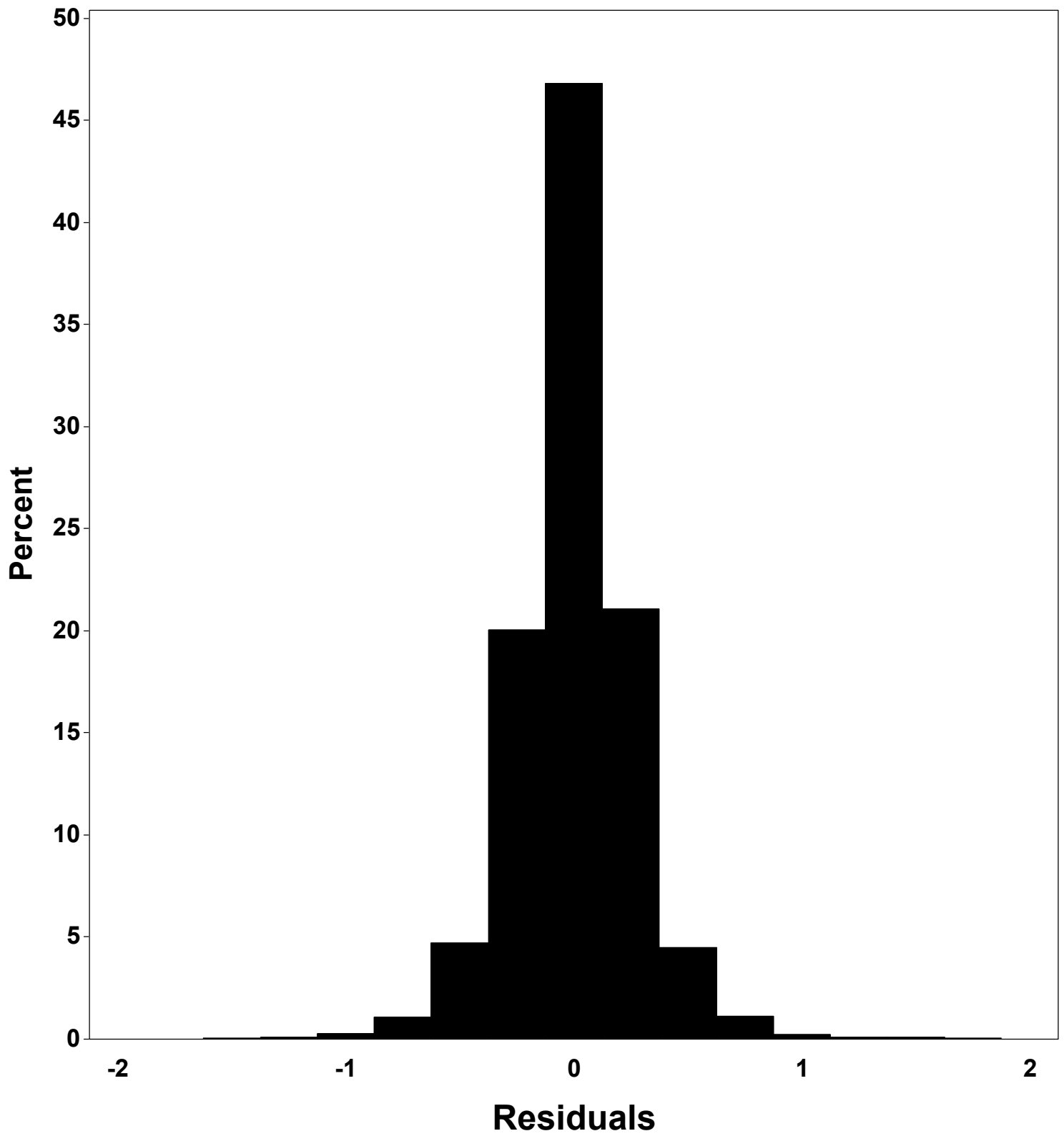

Supplement: S10 Fig — A histogram of the distribution of conditional BMI SDS residuals in girls, showing to be normal distributed among the girls. (PDF) [file pone.0120177.s010.pdf]

# Boys

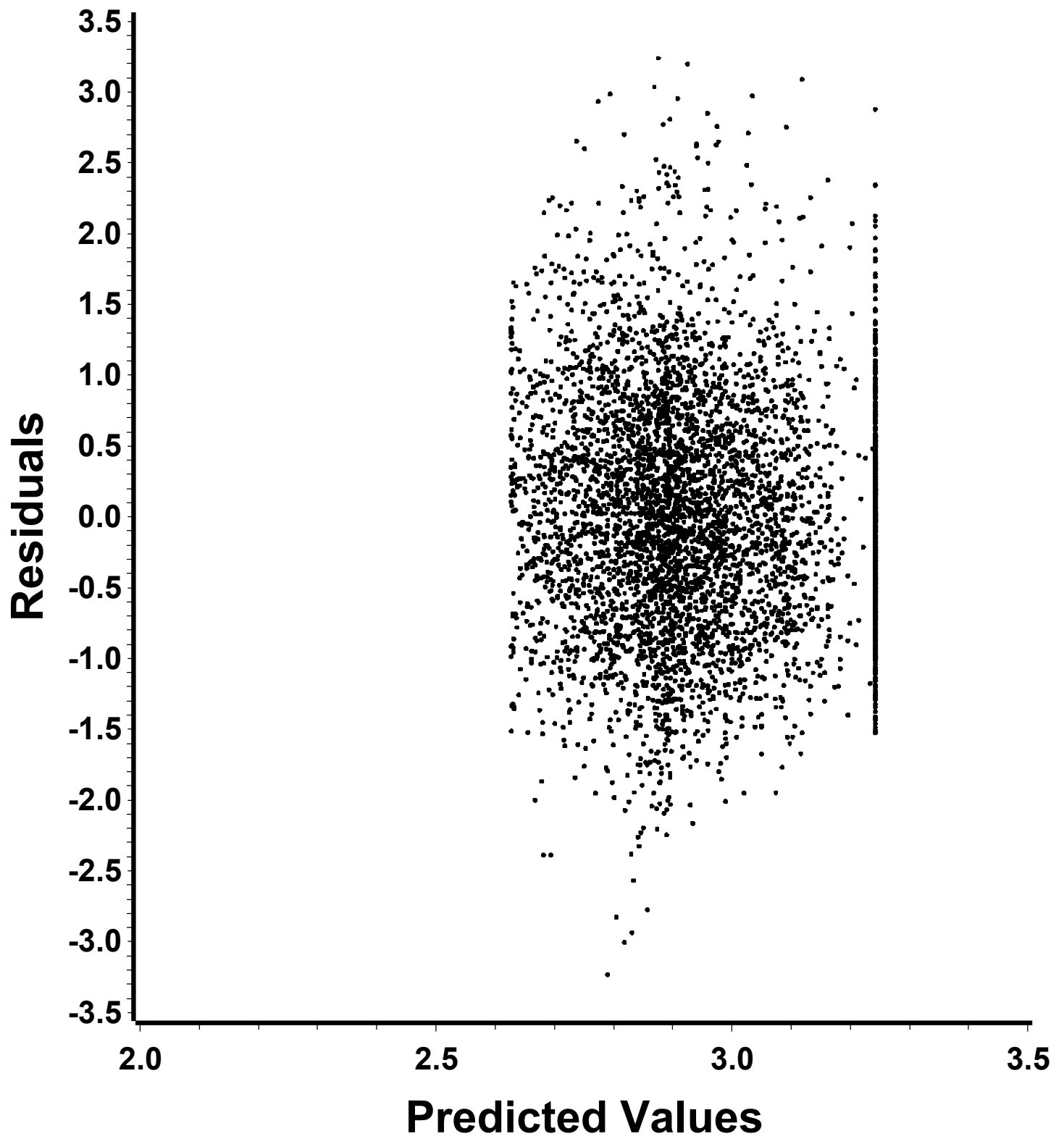

Supplement: S11 Fig — A plot of the conditional residuals in boys compared with the predicted values. (PDF) [file pone.0120177.s011.pdf]

# Girls

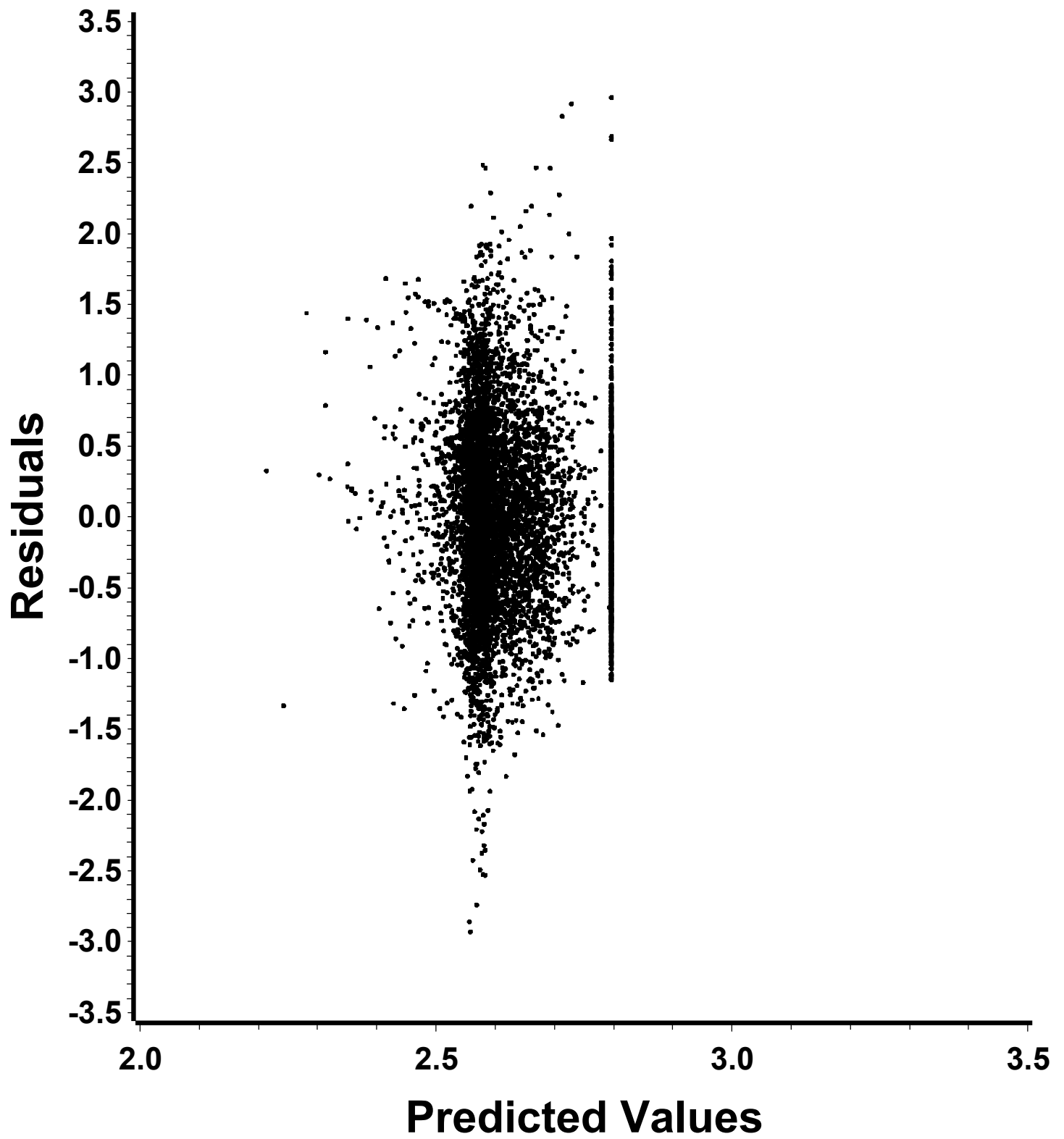

Supplement: S12 Fig — A plot of the conditional residuals in girls compared with the predicted values. (PDF) [file pone.0120177.s012.pdf]

# Boys

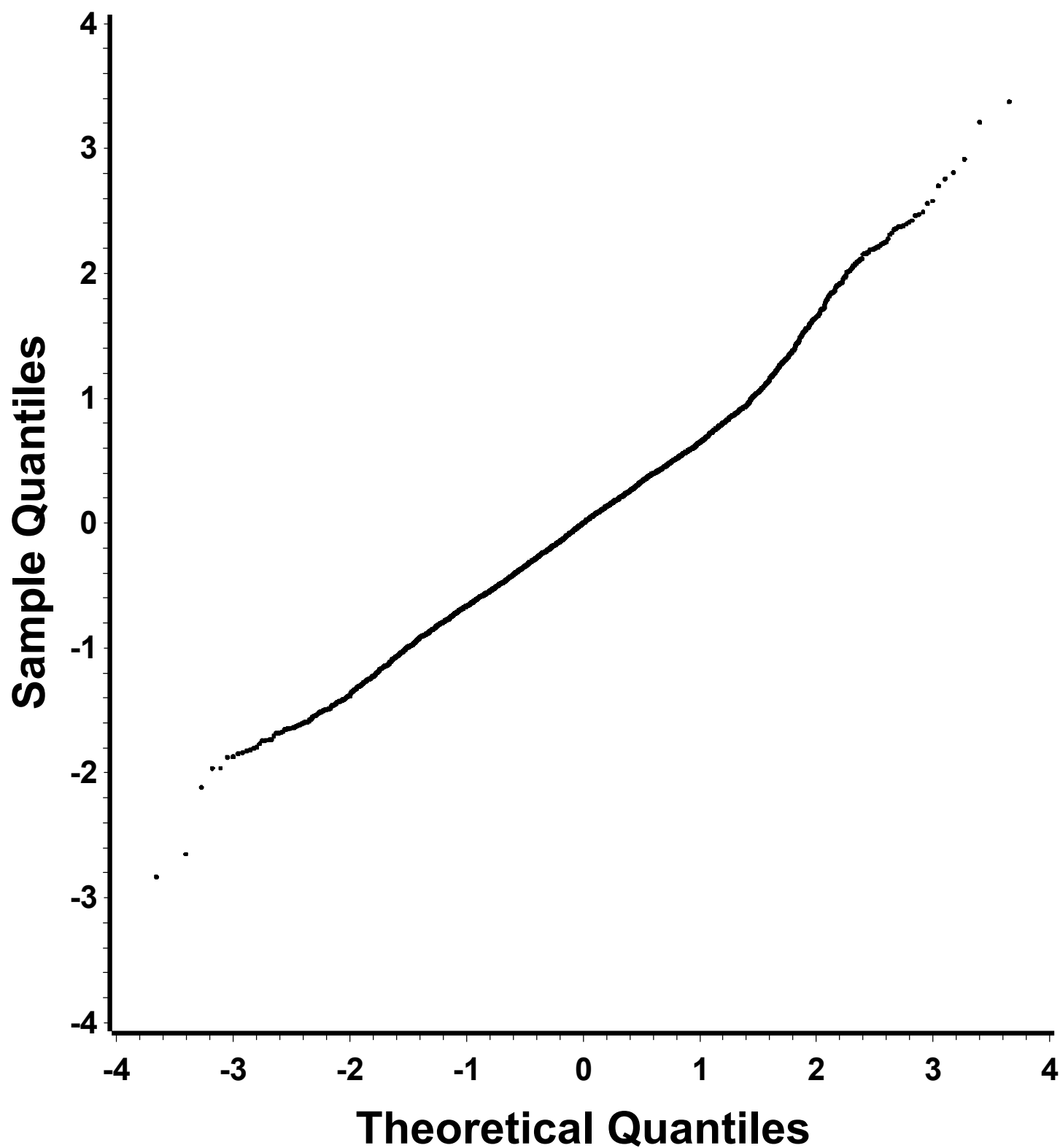

Supplement: S13 Fig — A plot comparing the sample quantiles with the theoretical quantiles of the conditional residuals in the boys. (PDF) [file pone.0120177.s013.pdf]

# Girls

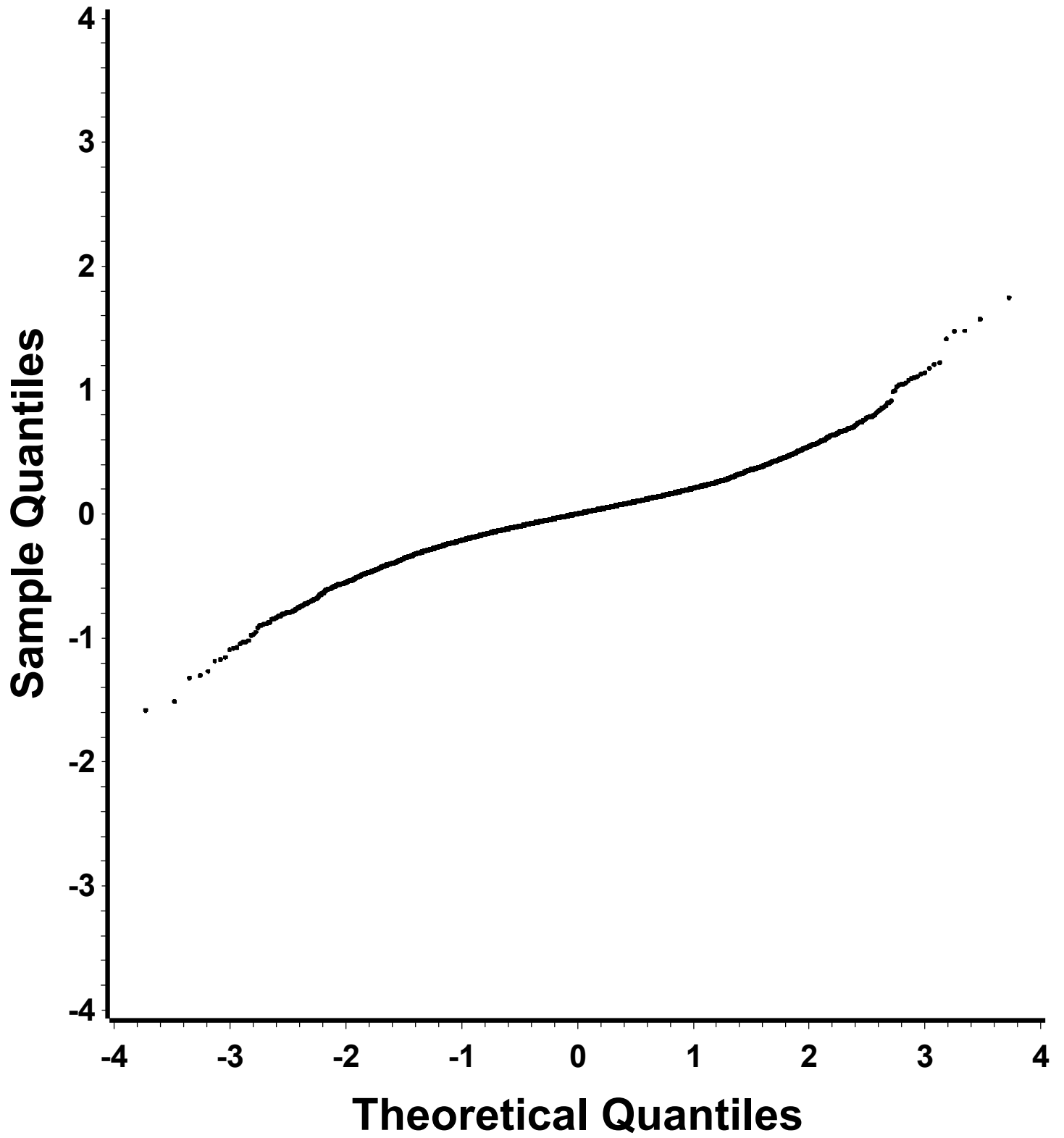

Supplement: S14 Fig — A plot comparing the sample quantiles with the theoretical quantiles of the conditional residuals in the girls. (PDF) [file pone.0120177.s014.pdf]

# Boys

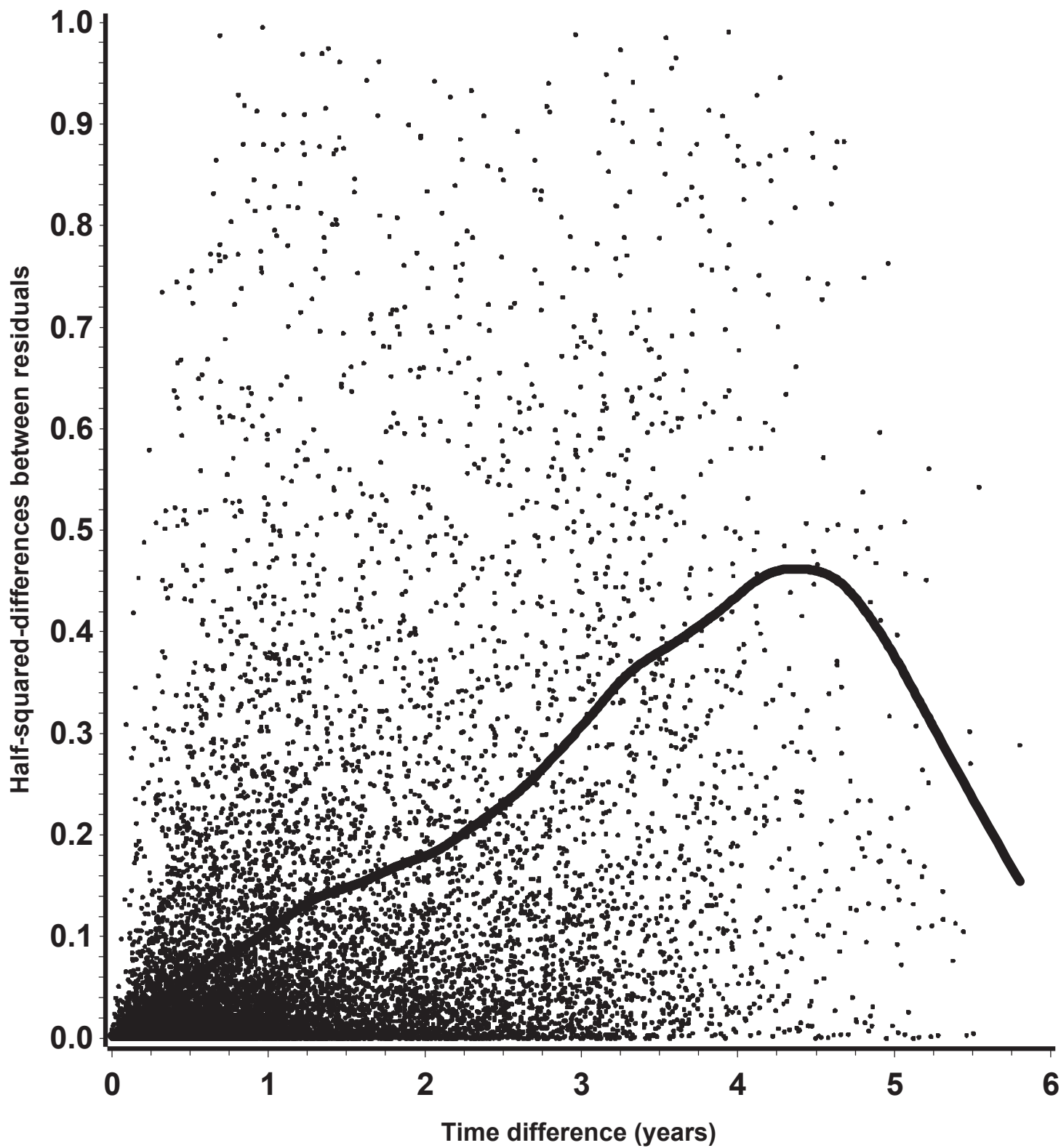

Supplement: S15 Fig — A plot of the half-squared differences between residuals over differences in time for the boys, with a smoothed curve showing that the covariance declines as time between measurements increases. (PDF) [file pone.0120177.s015.pdf]

# Girls

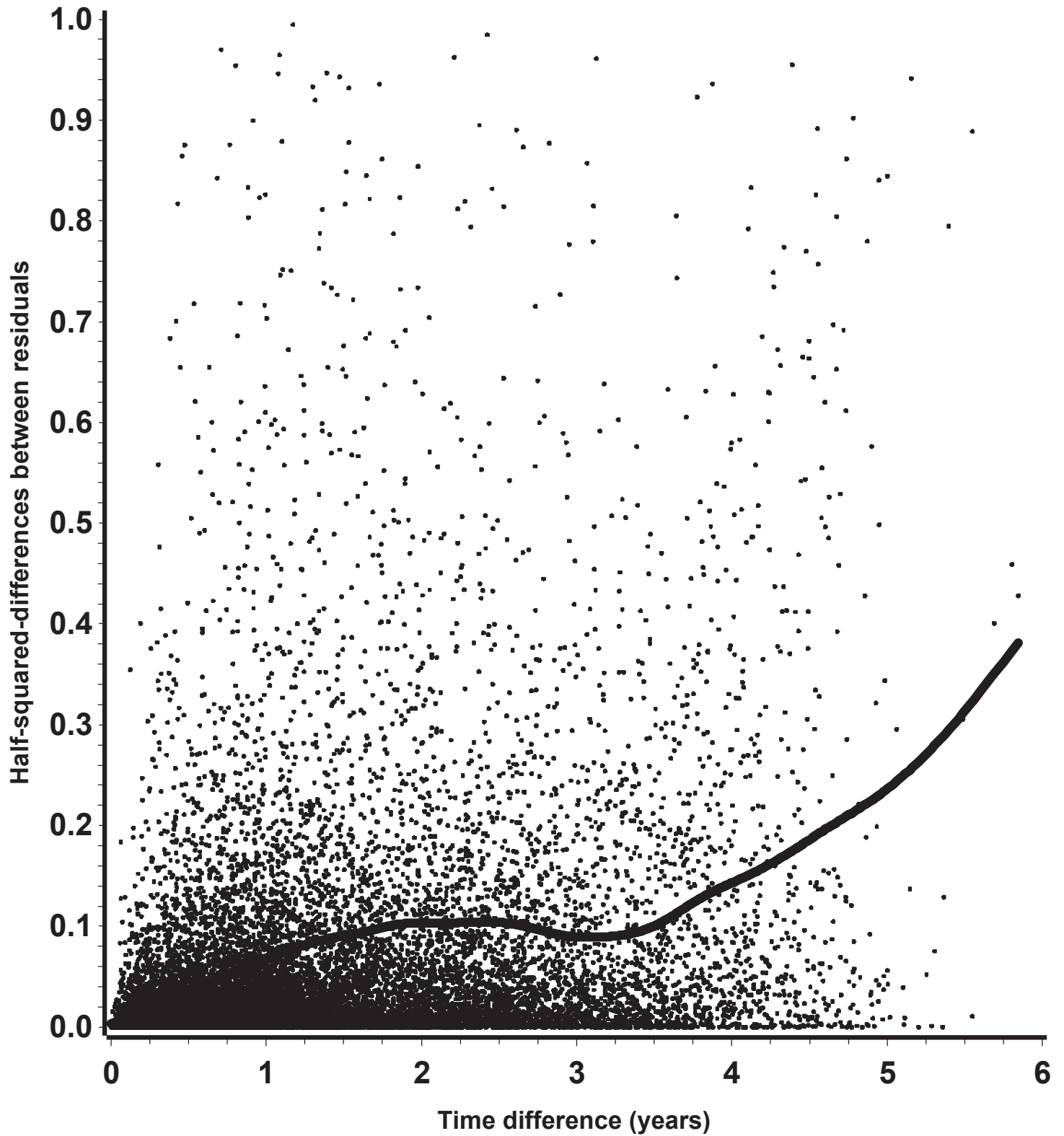

Supplement: S16 Fig — A plot of the half-squared differences between residuals over differences in time for the girls, with a smoothed curve showing that the covariance declines as time between measurements increases. (PDF) [file pone.0120177.s016.pdf]
